# Shapelet Discovery by Lazy Time Series Classification

**DOI:** 10.1155/2020/1978310

**Published:** 2020-10-24

**Authors:** Wei Zhang, Zhihai Wang, Jidong Yuan, Shilei Hao

**Affiliations:** School of Computer and Information Technology, Beijing Jiaotong University, Beijing 100044, China

## Abstract

As a representation of discriminative features, the time series shapelet has recently received considerable research interest. However, most shapelet-based classification models evaluate the differential ability of the shapelet on the whole training dataset, neglecting characteristic information contained in each instance to be classified and the classwise feature frequency information. Hence, the computational complexity of feature extraction is high, and the interpretability is inadequate. To this end, the efficiency of shapelet discovery is improved through a lazy strategy fusing global and local similarities. In the prediction process, the strategy learns a specific evaluation dataset for each instance, and then the captured characteristics are directly used to progressively reduce the uncertainty of the predicted class label. Moreover, a shapelet coverage score is defined to calculate the discriminability of each time stamp for different classes. The experimental results show that the proposed method is competitive with the benchmark methods and provides insight into the discriminative features of each time series and each type in the data.

## 1. Introduction

In recent years, massive time series data have been generated in many fields, including weather forecasting [1], malware detection [2], voltage stability assessment [3], human identification [4], and biomedicine [5]. Hence, the study of time series has been widely applicable, among which classification is an important research field. The classification issue of time series is the same as the traditional classification problem. We hope to find a function that can map any time series to a target class label. Although a large number of time series classification algorithms have been proposed, extensive experiments show that the 1NN classifier combining different distance metrics is still a competitive model in many problem areas [6–12]. In addition to the common Euclidean distance, alternatives have been proposed to measure the similarity between time series, including dynamic time warping (DTW) [13], weighted DTW (WDTW) [14], edit distance with real penalty (ERP) [15], time warp edit (TWE) [16], and move-split-merge (MSM) [17].

The improved distance function can help promote the performance of the nearest-neighbor model, but the 1NN classifier presents obvious drawbacks. This classifier cannot indicate the common characteristics of similar instances and the dissimilarity between different classes. In other words, its interpretability is insufficient. In reality, except for the accuracy, the features of distinct instances are our concern. These features provide a deeper understanding of data and improve the interpretability of the classification model. Unfortunately, time series usually have no definite features. Hence, various feature prototypes are proposed to mine potential patterns of time series [9, 18, 19]. Among them, the most classic is the local discriminative features “shapelet” [18].

**Figure 1:**
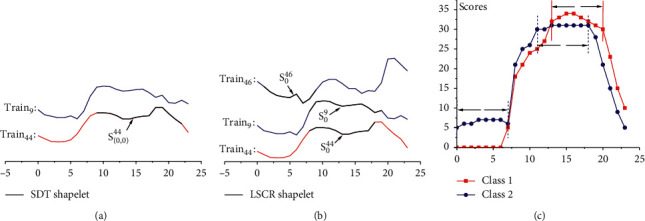
(a) The shapelet found by SDT for the ItalyPowerDemand dataset, and its corresponding training instances. The black bold part indicates the discovered time series shapelet. (b) Three time series from the ItalyPowerDemand training dataset and their corresponding shapelets captured by LSCR. Train_9_ and Train_44_ (in blue and red, respectively) are instances with different classes, while Train_9_ and Train_46_ (in blue) are instances of the same type Class2. Based on the discovered shapelets, our model can make correct predictions for the three instances. (c) The classwise shapelet coverage score obtained on the training set for each sampling point.

A shapelet (as shown in Figure 1(a)) is a special discriminatory subsequence of time series, which is originally applied to construct the shapelet-based decision tree (SDT) [18] through recursively searching for the best shapelet in the training set. Since the shapelet can be used to establish an interpretable classification model, it has been widely studied [9]. Shapelet-based classification models can be divided into two categories. One type of the method utilizes the top-*k* shapelets to create a transformed dataset, on which the traditional classification algorithms [20–24] could be applied. The other uses the shapelets to build the classification model directly [18, 25–28].

One major problem that all shapelet-based approaches generally face is the massive size of the candidate shapelet set. To solve this problem, researchers have put forward several methods. These methods can be roughly divided into four categories: (1) training instances are selected to generate the candidate shapelets. For example, Ji et al. [29] put forward a subclass splitting method to sample the training instances for candidate shapelet generation. (2) Heuristic shapelet search method. Grabocka et al. [27] presented a heuristic gradient descent shapelet search algorithm, which created a smaller candidate shapelet set. Rakthanmanon et al. [26] proposed a fast shapelet discovery algorithm based on Symbolic Aggregate approXimation (SAX). Similarly, Fang et al. [28] introduced a novel method to search shapelets based on piecewise aggregate approximation (PAA). (3) A random selection mechanism is used to select shapelets. Renard et al. [30] first proposed a random-shapelet algorithm to build the decision trees. Karlsson et al. [31] constructed the shapelet-based random forest, in which each decision tree is built based on instances and shapelets selected randomly. Further, to omit the shapelet threshold search, Shi et al. [32] have put forward the random pairwise shapelet forest. (4) Reformulating the shapelet search problem into a numerical optimization problem. For example, Hou et al. [33] treated the shapelet search task as a numerical optimization problem, and then the shapelets were learned by numerical analysis methods. Likewise, Wang et al. [34] designed a semisupervised shapelet learning model, which transforms the feature search problem into a joint optimization problem. Ma et al. [35] proposed an end-to-end model to learn the most discriminative shapelets by the gradient descent method. Zhao et al. [36] recently proposed a regularized shapelet learning framework to improve the shapelet learning efficiency. Although the above methods improve the classification efficiency of shapelet-based models to some extent, the vast majority of shapelet-based global classification models still has the following disadvantages:

1. The shapelets captured by most shapelet-based models cannot adequately reflect the information of feature distribution and frequency of each class in the dataset. For example, due to the existence of intraclass variation, a few instances in different classes may have low-frequency discriminative features.
2. The whole training set is generally applied for the discriminatory evaluation of candidate shapelets. Owing to the influence of the redundant instances and the intraclass variability, the extracted shapelets are merely the best on average for instances in the training dataset and cannot accurately reflect the local characteristics of the instance to be classified. In other words, the established shapelet-based model is not suitable or efficient for each test instance. Targeted evaluation strategies are not given enough attention.

To address these problems, we have first proposed a lazy shapelet-based model to capture the local features of each instance in the literature [37] (the literature [37] is a poster in the conference ICONIP 2018. In the conference manuscript, we simply proposed a lazy model to classify the instance based on its own local features. However, in this paper, the extended version further studies the fusion of global and local similarity, the local feature distribution and frequency information discovery, etc. In addition, this paper includes details about how to discover the local features for each instance to be classified in the shapelet-based model, how to use the local characteristics of each instance to determine the classwise discriminatory information, more experiments on parameter setting, statistical analysis, model comparison, and case studies). However, the proposed model still cannot get insight into the feature distribution and frequency information of the time series data. For that, we significantly extend the research on the data-driven, shapelet-based model (lazy shapelet classification route, LSCR) to study the feature distribution and frequency information discovery. Here, the advantages of our model are interpreted in conjunction with Figure 1. From Figure 1(b), it can be found that the heterogeneous instances are different, and that there are also differences between the homogeneous instances. Since LSCR performs targeted analysis for each instance, compared with the SDT shapelet (as shown in Figure 1(a)), our model may capture characteristics that SDT cannot discover. For example, the shapelet S_0_^46^ does not appear in the model built by SDT. Further, to evaluate the classwise discriminative feature frequency, the shapelet coverage score is defined. From Figure 1(c), we find that the scores can not only indicate the local discriminant intervals for different classes but also reflect their frequency information. For instance, the low-frequency discriminative interval [0, 7] reflects the location of the local features detected by our model on a few instances of Class2, which are usually caused by intraclass variation and ignored by the global shapelet-based model. The main contributions of this paper are summarized as follows:

1. In contrast with the classical *k*NN or 1NN model based on global similarity, our model is a fusion of global and local similarities. For the consideration of global similarity, the instance selection strategy is used in evaluating the discrimination of shapelets. The smaller evaluation dataset can eliminate the interference of intraclass variation and improve the classification performance. In addition, local similarity is applied instead of global similarity for prediction, which makes the proposed model more interpretable.
2. To reduce the massive number of redundant candidate shapelets generated by the brute-force algorithm, a novel strategy is proposed for extracting candidate shapelets from the instance to be classified. This strategy can guarantee that the extracted shapelets accurately reflect the local characteristics of each test instance.
3. The shapelet coverage score of each sampling point is calculated to analyze the local characteristics of different classes in the dataset. Since the proposed model can efficiently analyze the local features of each instance, more accurate local characteristic information can be obtained. In particular, the classwise discriminative feature frequency and distribution information can be presented, which can help us to understand the data more comprehensively.

The remainder of the paper is organized as follows.  Section 2 introduces related concepts and basic theories.  Section 3 describes the proposed model and algorithm design in detail.  Section 4 presents the experimental analysis.  Section 5 offers the conclusion of this paper.

## 2. Definitions and Notation

In this section, some definitions and formulas related to our model will be presented.

**Definition 1 .**

(time series). A time series *T* is an ordered sequence that contains *m* actual observation values *t*_1_, *t*_2_,…, and *t*_*m*_, i.e., *T*={*t*_1_, *t*_2_,…, *t*_*m*_}, *t*_*i*_ ∈ *R*. The symbol **D**={*T*_1_, *T*_2_,…, *T*_*n*_} represents the dataset containing *n* time series.

**Definition 2 .**

(time series subsequence and shapelet). Given a time series *T*={*t*_1_, *t*_2_,…, *t*_*m*_}, a subsequence *S* of *T* contains *l* consecutive values from *T*; that is, *S*={*t*_*i*_, *t*_*i*+1_,…, *t*_*i*+*l*−1_}, where 1 ≤ *i* ≤ *m* − *l* + 1. A shapelet is a tuple (*S*, *δ*) that consists of a subsequence *S* and a distance threshold *δ*.

**Definition 3 .**

(candidate shapelet set). The candidate shapelet set is composed of subsequences of time series.

The symbol **D**_node_ is a dataset corresponding to an arbitrary tree node in the model SDT or LSCR, and *W*_*l*_(**D**_node_) is a set of candidate shapelets with length *l* built on the dataset **D**_node_. *W*_*l*_(**D**_node_) in SDT could be represented as follows:

(1) $$\begin{array}{c} W_{l}\left(D_{\text{node}}\right)=\underset{T_{i}\in D_{\text{node}}}{\cup }W_{l}\left(T_{i}\right), \end{array}$$

where *W*_*l*_(*T*_*i*_) denotes the set of subsequences with length *l* from *T*_*i*_.

However, in our work, since the best shapelets are searched from the subsequence space of the instance to be classified, the set of shapelet candidates of length *l* for each node in LSCR is

(2) $$\begin{array}{c} W_{l}\left(D_{\text{node}}\right)=W_{l}\left(T\right), \end{array}$$

where *T* is the test instance.

Then, the whole candidate shapelet sets *W*(**D**_node_) for each node in SDT and LSCR can be obtained through the following equation:

(3) $$\begin{array}{c} W\left(D_{\text{node}}\right)=\overset{\text{max}}{\underset{l=\text{min}}{\cup }}W_{l}\left(D_{\text{node}}\right), \end{array}$$

where min and max are the minimum and maximum candidate lengths, respectively.

Therefore, compared with SDT, LSCR reduces the scale of candidate shapelets in a single node by an order of magnitude.

**Definition 4 .**

(similarity of equal-length time series). Let dist(*T*_*i*_, *T*_*j*_) be a similarity function of time series, which takes *T*_*i*_ and *T*_*j*_ with equal length as the input. The function will return a nonnegative value, which represents the similarity degree.

Generally, the smaller the distance is between two time series, the more similar the two time series are. In reality, we often need to judge the similarity between unequal time series. For example, in our work, we need to determine whether a time series contains a specific local feature through the distance between a subsequence and a whole time series.

**Definition 5 .**

(similarity of unequal-length time series). Let distul(*T*, *S*) be a similarity function, in which time series *T* and *S* have different lengths. The function returns a nonnegative optimal matching distance between two sequences as the degree of similarity. The distance between time series *T* and sequence *S* is

(4) $$\begin{array}{c} \text{distul}\left(T,S\right)=\min\left(\text{dist}\left(S_{i},S\right)\right),S_{i}\in W_{\left|S\right|}, \end{array}$$

where the symbol *W*_|*S*|_ represents the subsequence set with length |*S*| of time series *T* (|*S*| and |*T*| denote the length of sequences *S* and *T*, respectively, and  |*S*| ≤ |*T*|).

In our model, the distance between subsequences with equal length is calculated by the Euclidean distance, while the distance between complete time series is measured by the specified distance function.

In reality, different subsequences may have disparate discriminability. In our work, information gain is used to measure the discrimination of shapelets. To reduce distance computation and improve the shapelet discriminant property, the concept of the evaluation dataset is put forward.

**Definition 6 .**

(shapelet evaluation dataset). The shapelet evaluation dataset is a specific subset of the training dataset, which is designed to evaluate the discriminability of local features of each test case.

**Definition 7 .**

(entropy of dataset). The entropy of a given dataset **D** is calculated by the following formula:

(5) $$\begin{array}{c} H\left(D\right)=-\sum_{i=0}^{\left|C\right|-1}p\left(D_{c_{i}}\right)\log\left(p\left(D_{c_{i}}\right)\right), \end{array}$$

where *c*_*i*_ is an element in the class value set *C* of **D**, **D**_*c*_*i*__ is the subset of instances with class *c*_*i*_ in **D**, and the proportion *p*(**D**_*c*_*i*__) is calculated by

(6) $$\begin{array}{c} p\left(D_{c_{i}}\right)=\frac{\left|D_{c_{i}}\right|}{\left|D\right|}. \end{array}$$

**Definition 8 (shapelet information gain).**

Given a shapelet *S* and a dataset **D** containing instances with different classes, the information gain of *S* is calculated as follows:

(7) $$\begin{array}{c} \text{IG}\left(D,S\right)=\underset{s}{\text{arg}\max\left(H\left(D\right)\right)}-\;\frac{\left|D_{\text{left}}\right|}{\left|D\right|}H\left(\left|D_{\text{left}}\right|\right)-\frac{\left|D_{\text{left}}\right|}{\left|D\right|}H\left(\left|D_{\text{right}}\right|\right), \end{array}$$

where *s* is a split distance that can be applied to divide the dataset into two subsets: **D**_left_={*T*_*i*_|distul(*T*_*i*_, *S*) ≤ *s*} and **D**_right_={*T*_*i*_|distul(*T*_*i*_, *S*) > *s*}.

Finally, the maximum value of information gain is normally treated as the shapelet discrimination, and the corresponding distance *s* is taken as threshold *δ*. The split distance *s* usually takes the middle distance between two distance points. The detailed calculation process can be found in the literature [18]. In our model, the shapelet information gain calculated by equation (7) on a specific evaluation dataset reflects the reduction in uncertainty in the predicted class label of the test instance. Here, the mathematical description of our model is introduced.

Given a specific test instance *T*, its uncertainty of predicted class in our model is

(8) $$\begin{array}{c} U\left(T\right)=H\left(D_{e}\left(T\right)\right), \end{array}$$

where **D**_*e*_(*T*) is the initial evaluation dataset of *T*.

In the prediction process, the proposed model LSCR tries to progressively reduce the uncertainty by its own characteristics. The model can be formulated as

(9) $$\begin{array}{c} \underset{S}{\text{minmize}\left(U\left(T\right)\right)}=\underset{S}{\text{minimize}\left(\text{IG}\left(T,D_{e}\left(T\right)\right)\right)}-\sum_{i=0}^{\left|S\right|-1}IG\left(D{\left(T\right)}_{e}^{i},S_{i}\right), \end{array}$$

where *S*_*i*_ denotes the *i*th element in the learned shapelet set **S** of *T*.

Additionally, **D**(*T*)_*e*_^*i*^ is the corresponding evaluation dataset of *S*_*i*_, which is determined by

(10) $$\begin{array}{c} D{\left(T\right)}_{e}^{i}=\left\{\begin{array}{cc} D_{e}\left(T\right), & i=0,\\ \left\{\left.T^{\prime }\right|\text{dist}\left(T^{\prime },S_{i-1}\right)\leq S_{i-1}.\delta ,T^{\prime }\in D{\left(T\right)}_{e}^{i-1}\right\}, & i>0. \end{array}\right. \end{array}$$

Finally, the main class property of the dataset **D**(*T*)_*e*_^|**S**|−1^ would be taken as the predicted class value of *T*. Generally, there will be only one type of instance left in the final evaluation dataset.

## 3. Targeted Shapelet Extraction Technique

### 3.1. Overview

**Figure 2:**
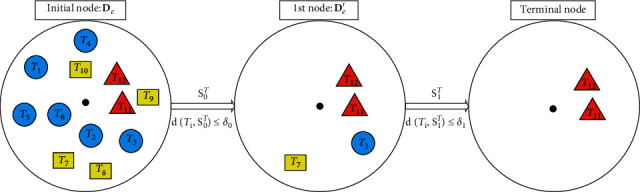
A shapelet classification route diagram with 3 nodes. The symbol $S_{i}^{T}$ denotes the *i*th shapelet on the classification route of the test instance *T*, and *δ*_*i*_ is its corresponding split threshold. The circle, rectangle, and triangle icons represent neighbors of *T* from Class1, Class2, and Class3, respectively. They are distributed around the center (the black spot) of the circle according to their distances from *T*.

To provide a brief introduction to the model LSCR, a schematic diagram is first presented in Figure 2. As determined from the figure, the evaluation dataset **D**_*e*_ for the test instance *T* is first generated; second, the candidate shapelets from *T* are evaluated on **D**_*e*_, and then the best shapelet is employed to exclude the instances that do not contain the local feature represented by S_0_^*T*^; third, the optimal shapelet on the dataset **D**_*e*_′ is continually searched until termination. Generally, there will be only one class of instances left in the final dataset, and the class will be taken as the predicted value.

The predicted result may be different from the class value of the nearest neighbor or the majority class in the initial dataset. This is the greatest difference between our model and the nearest-neighbor models. Here, the model will be described in detail.

### 3.2. Building Shapelet Evaluation Dataset

From the perspective of information theory, the purpose of extracting the best shapelet is to minimize the uncertainty of the class label of the test instance. The uncertainty is reflected in the class distribution of each subdataset generated based on whether the instance contains the specific feature. Hence, the more unbalanced the distribution of the subset of instances selected based on the shapelet, the more discriminative the feature. For example, for a binary classification problem, it is ideal to use the shapelet to divide the dataset containing instances with different class values into two subsets, each of which contains only one type of instance.

For the large-scale dataset, the running time of the searching shapelet is unbearable, so we attempt to sample the training instances for shapelet evaluation. In this paper, instances are selected based on the neighbor distance and the class value of the closest neighbor. Moreover, if the nearest-neighbor instances in the neighborhoods corresponding to the initial node of the classification path belong to the same class, then the route degrades into a single node. In particular, when the size of neighborhoods is set to 1, the model degenerates into 1NN, and the discriminant feature cannot be extracted effectively. In view of these problems, we propose to build a small targeted subset that contains instances with different classes for the instance to be classified. The subset ensures that the distinguishing nature of the local features can be evaluated. In addition, the data sampling strategy can eliminate the impact of intraclass variation on the local feature discriminant evaluation.

As shown in Algorithm 1, according to the class value of the nearest-neighbor instance, we select *k* identical and different instances for the test instance to construct a shapelet evaluation dataset (lines 2–6).

### 3.3. Finding the Optimal Shapelet

To reduce the computational complexity of extracting the best shapelet and make the extracted shapelets better reflect the characteristics of the test instance, a data-driven shapelet search algorithm is further proposed to find the best shapelet. We only search the best shapelets from the subsequence space of the instance to be classified so that the extracted shapelet accurately reflects the local features of each test instance. The process of generating the candidate shapelets collection for the test instance is given in Algorithm 2. In the algorithm, each subsequence of *T* with starting point *i* and length *j* constitutes the candidate shapelet set (line 4).

The candidate set corresponding to each node in the model SDT contains *O* (*nm*^2^) candidate shapelets, where *n* is the number of time series and *m* is the length of each time series. In our model, we only consider the subsequences of test instance *T*, so there are only *O* (*m*^2^) candidate shapelets to be evaluated for each node. Our work reduces the size of candidate shapelet collection of each node by one order of magnitude.

The purpose of extracting shapelets from time series is to classify the time series using the discriminator. The discriminability of shapelets provides us with a way to explain the classification results.  Algorithm 3 introduces the method for finding the best shapelet for the instance to be classified on the evaluation dataset. First, the candidate shapelet set is generated for *T* (line 3). Then, each candidate is evaluated by equation (7) to search for the best shapelet (lines 4–8).

The time complexity of our model to find the optimal feature is *O* (*km*^4^), while that of the brute-force search algorithm in SDT is *O* (*n*^2^*m*^4^). Considering that *k* is smaller than *n*, the calculation in the progress of searching for the best shapelet is significantly less. In addition, two ways to improve the efficiency of shapelet search are used [18, 25]: the early abandonment mechanism and the shapelet entropy pruning strategy.

### 3.4. Lazy Shapelet Classification Algorithm

A classification route based on shapelets for each instance to be classified is built through Algorithm 4.

Algorithm 4 mainly consists of five steps. First, the targeted shapelet evaluation dataset is established for *T* (line 1). Second, the candidate shapelet set is generated for *T* (line 2). Third, the model searches for the best shapelet on the evaluation dataset (line 3) and judges whether the termination condition is satisfied (line 4). It does not meet the termination condition at the initial node; that is, it will not degrade to the single node route. Generally, a best shapelet *S* is found. Fourth, the extracted shapelet *S* is applied to update the evaluation dataset for the child node (lines 7–8). Only the training instances whose distances are not greater than the split threshold are selected to form the subdataset. Fifth, the model repeats steps 2–8 until the termination condition is satisfied (line 10). Last, the shapelet-based classification route for *T* is returned (line 11).

### 3.5. Computing Shapelet Coverage Score

**Definition 9 (shapelet coverage).**

Shapelet coverage refers to the corresponding time interval of a given shapelet *S*. If a time stamp *t* falls within the shapelet coverage of *S*, then we state that *t* is covered by *S*.

In our work, the discriminatory score is calculated for each time stamp based on the coverage intervals of all obtained shapelets. First, an indicator function is presented to determine whether a time stamp *t* is covered by a given shapelet *S*:

(11) $$\begin{array}{c} I\left(t,S\right)=\left\{\begin{array}{cc} 1, & \text{if }t\in \left[S.sp,\;S.sp+|S|-1\right],\\ 0, & \text{otherwise,} \end{array}\right. \end{array}$$

where *sp* denotes the starting position of *S* in the time series.

Then, based on shapelets captured on the decision path of all correctly predicted time series, the importance of time stamps for different classes can be evaluated through the following formula:

(12) $$\begin{array}{c} \text{score}\left(t,c\right)=\sum_{T\in D_{\text{correct}}^{c}}\sum_{S\in S_{T}}I\left(t,S\right), \end{array}$$

where **D**_correct_^*c*^ represents the set of correctly predicted instances with class value *c* and **S**_*T*_ indicates the set of shapelets captured on the classification path of *T*.

In essence, the coverage score score(*t*, *c*) reflects the discriminability of the time stamp *t*, which can be used to detect the distribution of distinguishing feature intervals and the feature frequency information for each category. Generally, the interval composed of several consecutive time stamps with similar scores corresponds to the local differentiating features. Therefore, in our work, the interval satisfying the condition would be regarded as the local feature location. In addition, the scores demonstrate their occurrence frequency.

Here, the algorithm of computing the shapelet coverage scores for different classes in the dataset will be introduced.

In Algorithm 5, to compute the shapelet coverage score for each class, the dataset **D**_correct_^*i*^ of the correctly predicted instances with the specific class is first obtained (lines 2-3). Then, all shapelets captured by LSCR for every instance in **D**_correct_^*i*^ are collected (lines 4–6). Finally, the scores reflecting the discriminability of each time stamp for every class are calculated based on equation (12) (line 7).

## 4. Experiments

Experimental analyses are conducted on 20 datasets from the UCR time series repository [38], most of which are frequently used for evaluation of shapelet-based models [9, 21, 22, 24, 27]. The experimental data are divided into training and test parts. The former part is used to build the model, while the latter part is applied to calculate the classification accuracy. The information of datasets is listed in Table 1, including train (size of the training set), test (size of the test set), max_*k* (the minimum number of instances of a class in the training set; that is, the maximum value that parameter *k* can take.), length (the length of time series), and classes (the number of classes).

### 4.1. Parameter *k* Analysis

To study the effect of the size of the evaluation dataset on the discriminative evaluation of shapelets, the accuracy trends of LSCR_DTW_ within the specified range over 10 datasets are first analyzed as a representative. Then, the average accuracy curves of 5 fusion models (LSCR_DTW_, LSCR_ERP_, LSCR_ED_, LSCR_TWE_, and LSCR_MSM_) are presented for parameter setting.

**Figure 3:**
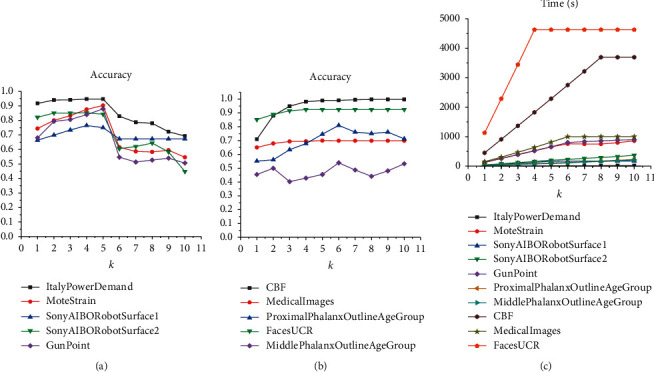
The accuracy and time variations of the model LSCR_DTW_ on 5 binary-class and 5 multiclass datasets with the value *k* increasing.

Figure 3 shows the sensitivity of prediction results of different datasets to parameter *k*. From Figure 3(a), it can be seen that most accuracy rates on 5 binary-class datasets reach the maximum values when parameter *k* is 5, and then all of the curves show a significant downward trend. From Figure 3(b), except for the MiddlePhalanxOutlineAgeGroup, the accuracy rates on 4 multiclass datasets exhibit a growth trend as *k* increases in the previous stage. When *k* is greater than 6, the accuracy on each dataset tends to be stable or decrease. The above experimental results suggest that the accuracy of LSCR generally varies regularly with *k*. Therefore, we propose to set *k* based on the trend of average accuracy.

**Figure 4:**
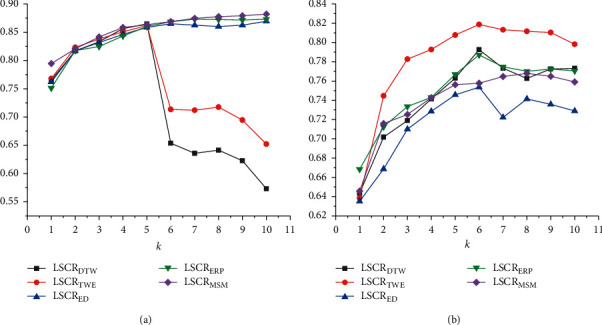
The average accuracy variations of the model LSCR_dist_ combined with different distance functions on (a) 5 binary-class and (b) 5 multiclass datasets with the parameter *k* increasing.

In Figure 4(a), it can be seen that, on binary-class datasets, the average accuracy variations of 5 fusion models show two significantly different trends. One shows a trend of increasing and gradually becoming stable, while the other presents a significant decline in accuracy after passing the inflection point. In Figure 4(b), on multiclass datasets, the average accuracies of all fusion models first increase and then decrease slowly after reaching the peak. Finally, in our work, the value *k* corresponding to the highest average accuracy is set as the final parameter of LSCR_dist_ on binary-class and multiclass datasets, respectively. See Table 2 for specific settings of the 5 fusion models.

**Figure 5:**
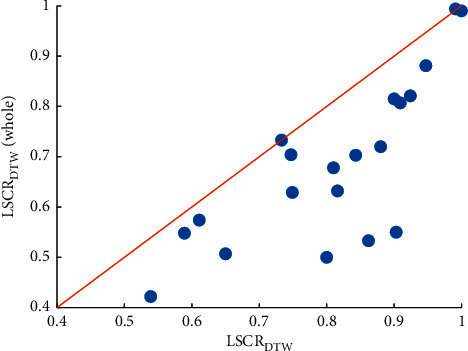
Accuracy comparison between LSCR_DTW_ combining instance selection for shapelet evaluation and LSCR_DTW_ (whole) using the whole training set as the evaluation set on 20 datasets.

In addition, the effect of instance selection on model performance is interpreted in Figure 5. In the scatter diagram, each point stands for a dataset. The more points there are in the figure that fall below the diagonal line, the better the performance of LSCR_DTW_. Since the targeted evaluation dataset is very helpful to improve the feature quality, the proposed shapelet evaluation strategy can significantly improve the performance of our model. As shown in Figure 5, LSCR_DTW_ outperforms LSCR_DTW_ (whole) on almost all datasets. The accuracy rates of the above two models are listed in Table 3.

### 4.2. Fusion Strategy Analysis

In this section, the effectiveness of the fusion strategy of global and local similarities is analyzed. Table 3 presents the accuracies of 5 1NN models combined with different distance functions (DTW, ERP, ED, TWE, and MSM) and their corresponding five fusion models. The accuracies of the 1NN models are taken from the website [38].

**Figure 6:**
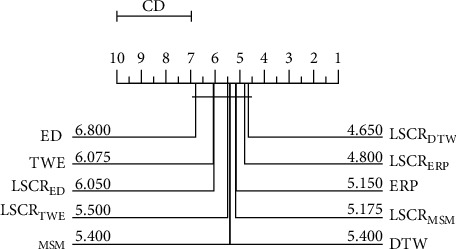
The critical difference diagram for 5 fusion models and 5 corresponding 1NN models on 20 datasets.

Figure 6 shows a critical difference diagram studied in literature [39, 40] for the 10 classification models on 20 datasets. This diagram is used for the overall test of significance of average ranks and can group models without significant differences into cliques. From the figure, we find that there are no significant differences in the performance of the 10 models on the 20 datasets, but the rankings of all fusion models are better than those of the corresponding 1NN models. This result suggests that the fusion model can effectively improve the classification performance of the 1NN model to some extent. Since LSCR_DTW_ ranks first, it will be used for further analysis in the following.

### 4.3. Performance Analysis

In this section, the proposed model is compared with shapelet-based classifiers and 1NN classifiers combining with many commonly used distance functions and deep learning models.

#### 4.3.1. Comparison with Shapelet-Based Classifiers

Since the classification process of the model proposed is similar to the decision tree, for the sake of fairness, all shapelet-based comparison models choose the decision tree to make predictions. Our model is first compared with SDT, fast shapelet tree (FS) [26], and C4.5 model [41] based on two respective shapelet transform algorithms. The symbol SSC4.5 denotes the C4.5 model based on the transform algorithm proposed by Yuan et al. [22], and STC4.5 represents the C4.5 model based on the other one proposed by Lines et al. [20]. The shapelet length ranges from 3 to the full length of time series and increases by 1 each time. All experimental results are listed in Table 4, and the accuracies of FS are obtained from the website [38]. In addition, the last line in Table 4 provides the average accuracy of each model over 20 datasets.

**Figure 7:**
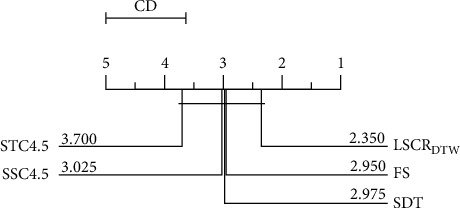
The critical difference diagram for LSCR_DTW_ and 4 shapelet-based models on 20 datasets.

As observed from Figure 7, it is clear that although there is no significant difference between LSCR_DTW_ and the other 4 shapelet-based models, the average rank of LSCR_DTW_ is the best.

**Figure 8:**
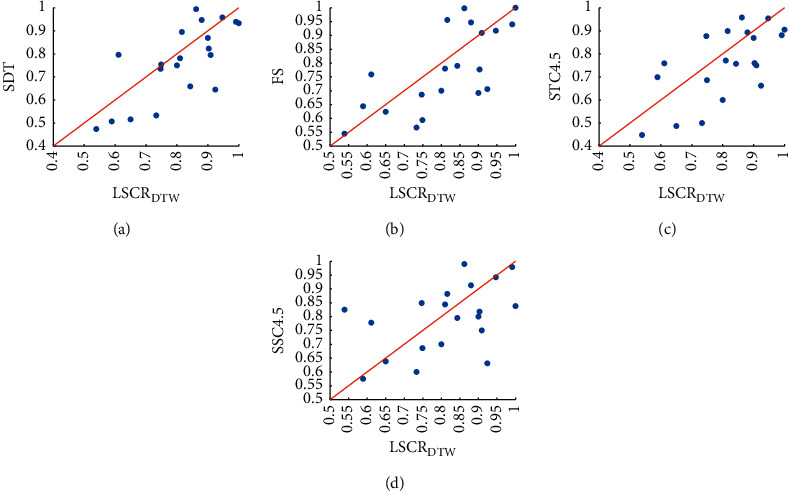
Accuracy comparison between LSCR_DTW_ and 4 shapelet-based classifiers.

Figure 8 presents the scatter plot of accuracy comparison between LSCR_DTW_ and the 4 classical shapelet-based classifiers.  Figure 8(a) shows that LSCR_DTW_ is better than SDT (14 of 20) over the 20 datasets. From Table 4, we find that compared with that of SDT built on the entire training set, the accuracies of LSCR_DTW_ on datasets MoteStrain, FacesUCR, SonyAIBORobotSurface2, etc., are significantly improved. In particular, on the dataset FacesUCR, the accuracy of LSCR_DTW_ is 20% greater than that of SDT. In Figures 8(b)–8(d), it can be concluded that LSCR_DTW_ is also better than STC4.5 (13 of 16), SSC4.5 (13 of 20), and FS (14 of 20). Hence, Figure 8 demonstrates that LSCR_DTW_ outperforms the existing shapelet-based decision tree model on the 20 datasets.

**Figure 9:**
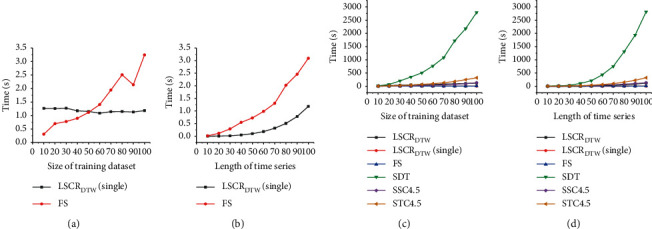
The running time trends of 6 shapelet-based models with increasing training dataset size and instance length, respectively.

To compare the time complexity of LSCR_DTW_ with other shapelet-based models, the changes in running times of 6 shapelet-based models with increasing number of instances and instance length are shown in Figure 9, respectively. LSCR_DTW_(single) represents the training time of a single model LSCR_DTW_ for a specific test instance, while LSCR_DTW_ represents the model running on the entire test set. In the experiment, a binary-class dataset with uniform distribution is designed to run the analysis models, and the size of the training set is always the same as that of the test dataset. For the first experiment, the sizes of training and test datasets increase by 10 at a time. The length of all instances remains 100. For another, the instance length increases by 10 at a time. The sizes of training and test sets are 100.

From Figures 9(a) and 9(b), we can determine that the training time of LSCR_DTW_ (single) is not sensitive to the size of the training set and is polynomial with respect to the length of the time series. In particular, it is faster than the current fastest shapelet-based model FS. Further, as observed from Figures 9(c) and 9(d), with increasing training set size and instance length, the time consumption gap between SDT, STC4.5, and LSCR_DTW_ widens, while the gap between LSCR_DTW_ and SSC4.5 is not obvious.

In conclusion, LSCR_DTW_ is an accurate and rapid shapelet-based classification model, which is the basis of learning feature distribution and frequency information.

#### 4.3.2. Comparison with Various 1NN Classifiers

In this section, except for the five distance functions listed above, we further compare our model with several DTW variants, including WDTW, complexity-invariant DTW (CID) [42], and derivative distance_DTW_ (DD_DTW_) [43]. The accuracies for the above 1NN models are provided by the website [38] (see Table 4).

**Figure 10:**
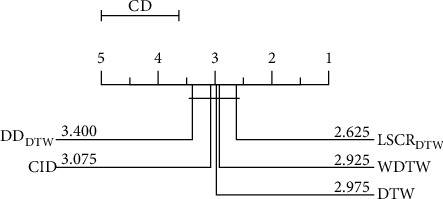
The critical difference diagram for LSCR_DTW_, DTW, and 3 DTW variants on 20 datasets.

**Figure 11:**
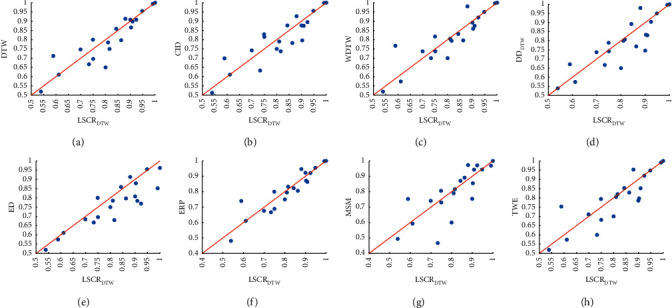
Accuracy comparison between LSCR_DTW_ and 8 1NN classifiers based on different distance functions.

Figure 10 demonstrates that our model exhibits no significant difference from DTW and its variants, but the average ranks of LSCR_DTW_ are the best. In Figure 11, it is clear that LSCR_DTW_ is better than ED (16 of 20), TWE (14 of 20), ERP (13 of 20), MSM (12 of 20), DTW (11 of 20), WDTW (14 of 20), CID (11 of 20), and DD_DTW_ (13 of 20).

In summary, LSCR_DTW_ is competitive with the 1NN model that depends on global similarity. The comparison results suggest that continuously narrowing the search space of class attributes based on local similarity is an effective way to make prediction.

#### 4.3.3. Comparison with Deep Learning Models

Now, various deep learning models have been widely studied in the field of time series classification. Karim et al. [44] attempted to improve the univariate time series classification performance of fully convolutional neural networks (FCNs) by using the long short-term memory recurrent neural network (LSTM-RNNs) submodules and attention mechanism and proposed the excellent models LSTM-FCN and ALSTM-FCN. Furthermore, the authors applied the above two models to the multivariate time series classification problem [45] and studied the reasons why the two models have superior performance [46]. Fawaz et al. [47] have reviewed some deep learning models of time series classification. Here, our model is compared with LSTM-FCN and 9 deep learning models (ResNet, FCN, Encoder, MLP, Time-CNN, TWIESN, MCDCNN, MCNN, and t-LeNet) analyzed in the literature [47]. All the experimental results were obtained from the corresponding literature (see Table 5).

**Figure 12:**
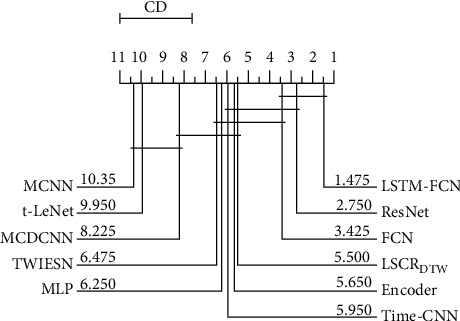
The critical difference diagram for LSCR_DTW_ and 10 deep learning models on 20 datasets.

**Figure 13:**
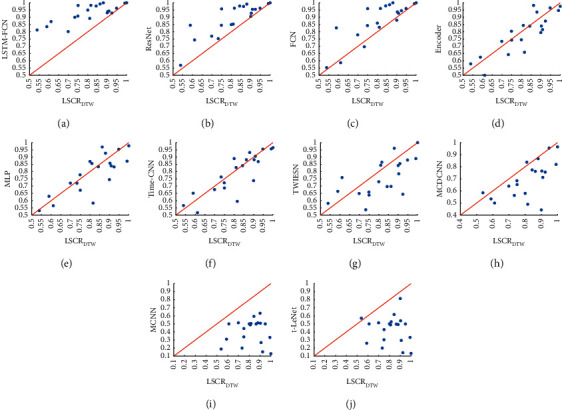
Accuracy comparison between LSCR_DTW_ and 10 deep learning models.

Figure 12 shows that, except for ResNet and FCN, LSTM-FCN is significantly better than other models, and that our model is significantly better than MCNN and t-LeNet. Among the 10 deep learning models, the average rank of our model is better than 7 of them. In Figure 13, it can be seen that LSCR_DTW_ is better than Encoder (12 of 20), Time-CNN (12 of 20), MLP (12 of 20), TWIESN (13 of 20), MCDCNN (17 of 20), t-LeNet (19 of 20), and MCNN (20 of 20).

Generally, to improve performance, deep learning model tuning requires an enormous computational cost. To pursue the optimal accuracy rate, Fawaz et al. [48] even proposed the neural network ensemble model with 60 deep learning models, but it is still not better than the traditional ensemble model HIVE-COTE [49]. It is unfair to compare our model with the deep learning model based on accuracy alone. In addition to improving accuracy, we believe that the model interpretability and data comprehensibility require more attention. However, the existing feature extraction methods for time series usually cannot simultaneously obtain the feature distribution and frequency information. For the deep learning model, it is difficult to train a targeted model for the specific instance on a small dataset. Accordingly, in our work, a highly interpretable classification model based on the lazy learning strategy is built for each target instance, which can be applied to gain insight into the local feature distribution and frequency information. The following is a detailed introduction.

### 4.4. Interpretability

To demonstrate the stronger interpretability of our model, this section separately analyses LSCR_DTW_ on a binary-class dataset, MoteStrain, and the CBF multiclass dataset.

#### 4.4.1. MoteStrain Dataset

Sensing data in MoteStrain are originally collected to detect potential variables online in the sensor network [50]. The classification task on this dataset is to distinguish whether the sensor is used for humidity measurement or temperature measurement. The classification performance of our model is significantly better than that of the model used for comparison and is close to the current best classification result provided by Bagnall et al. [38] on this dataset.

**Figure 14:**
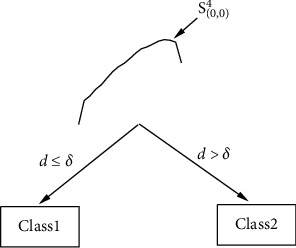
The shapelet decision tree and the shapelets found by SDT.

To further investigate the proposed model, the shapelet decision tree built by SDT is shown in Figure 14, where the symbol S_(0,0)_^4^ represents the shapelet extracted from the fourth training instance Train_4_ in the root node of the shapelet decision tree. As seen from Figure 14, there is only one shapelet in the decision tree, where *d* denotes the distance between the test instance and the shapelet and *δ* represents the split threshold of the shapelet. Based on the shapelet decision tree, when the distance between the test instance and the shapelet corresponding to the root node is not greater than the split threshold, the class prediction value of the test instance is Class1. Otherwise, it is Class2.

**Figure 15:**
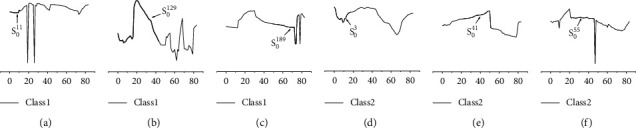
Six test instances from the MoteStrain dataset and their shapelets found by LSCR_DTW_. The three instances (a–c) are from Class1, and the other three (d–f) are from Class2. (a) Test_11_, (b) Test_129_, (c) Test_189_, (d) Test_3_, (e) Test_41_, and (f) Test_55_.

Figure 15 shows six instances and their shapelets extracted through LSCR_DTW_. Our model can correctly predict the class properties of these six instances, while SDT fails. Meanwhile, it is obvious that there are not only significant differences between instances of different types but also intraclass variations among similar instances. For example, the differences among the three instances with the same class label, Test_11_, Test_129_, and Test_189_, are noticeable, while there are no obvious common features. However, in the shapelet decision tree built by SDT, only a shapelet is found, which is not sufficient to distinguish the two classes. In contrast to the illustration of the shapelet given in Figure 14, it is not difficult to find that the optimal shapelet obtained in the shapelet decision tree is not the most discriminatory feature for the test instances. This result verifies that the shapelets extracted from the entire dataset are the most discriminatory for each training instance in the average sense; this is also the reason for the poor performance of SDT on the MoteStrain dataset.

Since the characteristics of each test instance have been considered in our model, in light of this situation, we can achieve better classification results. In addition, based on the shapelet obtained by LSCR_DTW_, the prediction process of each instance can be explained. For example, as shown in Figure 15, the reason why the 11th test instance belongs to Class1 is that the local feature S_0_^11^ lies in its initial stage, while the local feature S_0_^41^ of the 41st test instance in the middle part determines its predicted class label.

**Figure 16:**
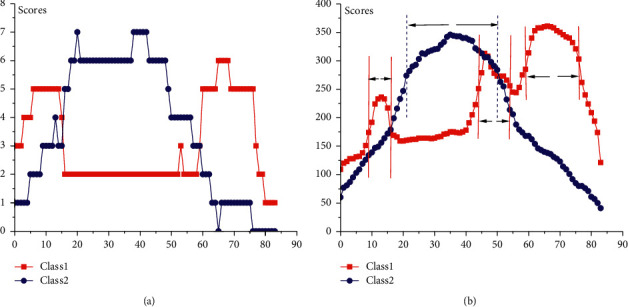
The shapelet coverage scores obtained on training and test datasets of MoteStrain: (a) training dataset and (b) test dataset.

Figure 16 displays the scores of shapelets coverage on the MoteStrain training and test datasets. It is obvious that the high score intervals on training and test sets are very similar. Since there are only 20 instances in the training set, the scores of shapelet coverage (as shown in Figure 16(a)) may not accurately reflect the local characteristics. However, we propose to directly evaluate the local characteristics of test cases, which can help us utilize large amounts of test data. In Figure 16(b), it can be seen that local features with different coverage frequencies of various classes have been detected from the test dataset, which cannot be captured by other shapelet-based models. For example, the intervals [9, 16] (low frequency), [44, 54] (medium frequency), and [59, 76] (high frequency) (as shown in Figure 16(b)) are three significant discriminative intervals of Class2, while the interval [21, 50] (high frequency) is the most discriminative part of Class1. Based on the proposed model, using more instances with accurate labels results in obtaining more accurate local feature information. The statistical information helps us acquire more comprehensive local characteristic information of time series data, such as feature distribution and frequency.

#### 4.4.2. CBF Dataset

**Figure 17:**
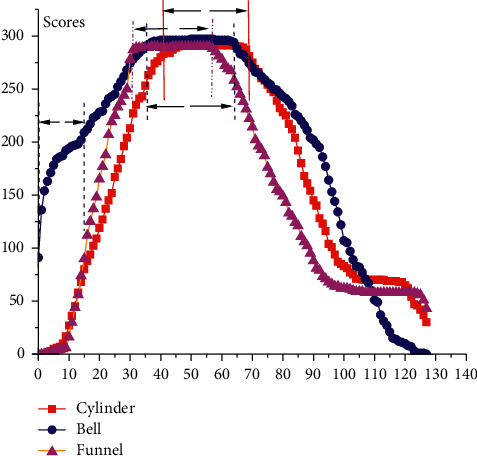
The shapelet coverage scores obtained on the CBF test dataset.

This section studies the multiclass dataset CBF, which contains three types: Cylinder, Bell, and Funnel.  Figure 17 shows the coverage scores of the CBF test dataset. It can be determined that the interval [31, 75] covers the most discriminative intervals for all three classes. In addition, unlike Cylinder and Funnel, the local interval [0, 15] with relatively low coverage frequency is discriminative for Bell.

**Figure 18:**
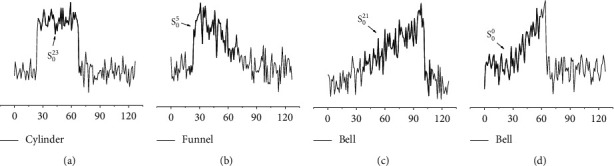
Four test instances from the CBF dataset and their shapelets found by LSCR_DTW_. The first two instances (a-b) are from Cylinder and Funnel, while the last two (c-d) are from Bell. (a) Test_23_, (b) Test_5_, (c) Test_21_, and (d) Test_0_.

Next, four specific test instances are presented. As shown in Figure 18, for the multiclass dataset, our model can not only detect high-frequency shapelets (as shown in Figures 18(a)–18(c)) but also effectively capture low-frequency shapelets (as shown in Figure 18(d)). The proposed model LSCR_dist_ is helpful to make a targeted analysis of each category of data and each test instance.

## 5. Conclusions

Aiming at the problems of global shapelet-based models built on the whole training set, a data-driven model fusing global and local similarities is proposed. In the model, the shapelet discriminability is evaluated through a specific subdataset. A smaller evaluation dataset reduces the computational time and improves the quality of shapelets. Moreover, target learning for each instance helps us understand the prediction process clearly. For example, the shapelets extracted by our LSCR model can be directly used to explain what characteristics determine the class value of the test instance. Furthermore, the proposed shapelet coverage score is applied to accurately analyze the local feature information of each class, which provides comprehensive insight into data characteristics. In the future, the application of the model in specific fields will be further studied, including ECG detection and image contour feature discovery.

## Figures and Tables

**Algorithm 1:**
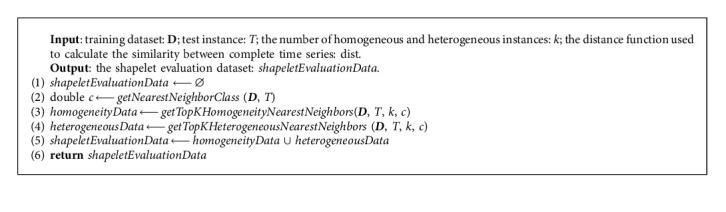
GetEvaluationDataFor*T* (**D**, *T*, *k*, dist).

**Algorithm 2:**
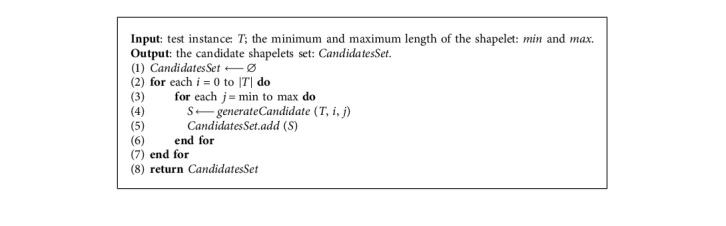
GenerateCandidatesFor*T* (*T*, min, max).

**Algorithm 3:**
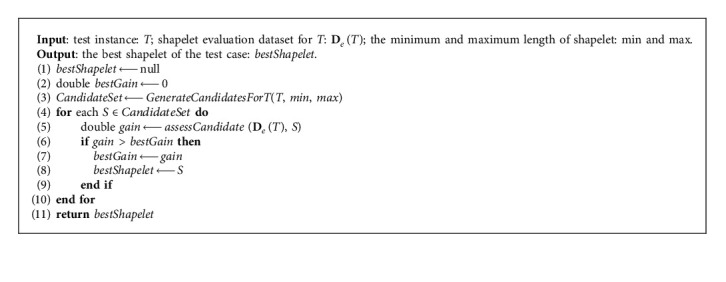
FindingBestShapelet (*T*, **D**_*e*_(*T*), min, max).

**Algorithm 4:**
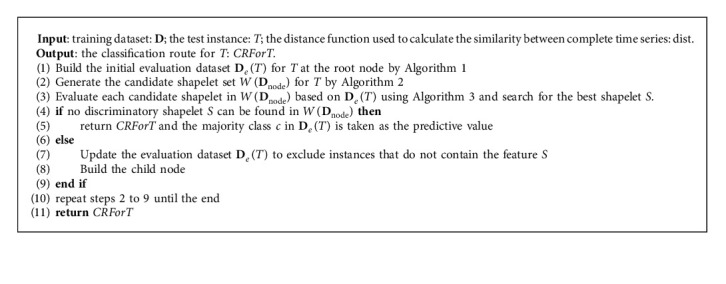
LSCR_dist_ (**D**, *T*).

**Algorithm 5:**
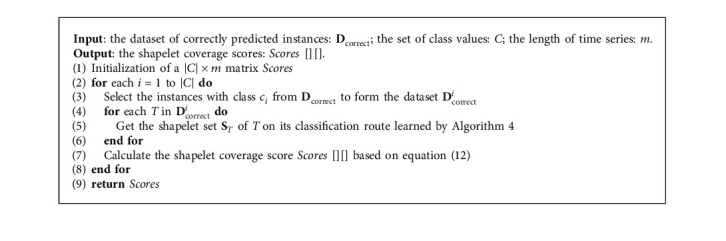
ComputeScore (**D**_correct_, *C*, *m*).

**Table 1:** Introduction of the experimental datasets.

Dataset	Train	Test	max_*k*	Length	Classes
ArrowHead	36	175	12	251	3
Beef	30	30	6	470	5
BeetleFly	20	20	10	512	2
CBF	30	900	8	128	3
ECGFiveDays	23	861	9	136	2
FaceFour	24	88	3	350	4
FacesUCR	200	2050	4	131	14
GunPoint	50	150	24	150	2
ItalyPowerDemand	67	1029	33	24	2
Lightning7	70	73	8	319	7
MedicalImages	381	760	6	99	10
MiddlePhalanxOutlineAgeGroup	400	154	55	80	3
MoteStrain	20	1252	10	84	2
Plane	105	105	9	144	7
ProximalPhalanxOutlineAgeGroup	400	205	72	80	3
SonyAIBORobotSurface1	20	601	6	70	2
SonyAIBORobotSurface2	27	953	11	65	2
ToeSegmentation1	40	228	20	277	2
ToeSegmentation2	36	130	18	343	2
Wine	57	54	27	234	2

**Table 2:** Parameter settings of all fusion models.

	LSCR_ED_	LSCR_DTW_	LSCR_TWE_	LSCR_MSM_	LSCR_ERP_
Binary	10	5	5	10	7
Multi	6	6	6	8	6

**Table 3:** The accuracies of LSCR_DTW_ (whole), 5 fusion, and 1NN models.

Dataset	LSCR_DTW_	DTW	LSCR_ERP_	ERP	LSCR_ED_	ED	LSCR_TWE_	TWE	LSCR_MSM_	MSM	LSCR_DTW_ (whole)
ArrowHead	0.749	0.800	0.749	0.800	0.794	0.800	0.743	0.794	0.726	0.806	0.629
Beef	0.733	0.667	0.667	0.667	0.700	0.667	0.733	0.600	0.700	0.467	0.733
BeetleFly	0.800	0.650	0.700	0.750	0.750	0.750	0.800	0.700	0.700	0.600	0.500
CBF	0.991	0.994	0.993	0.998	0.984	0.852	0.989	0.991	0.997	0.969	0.994
ECGFiveDays	0.863	0.797	0.905	0.806	0.880	0.797	0.879	0.829	0.900	0.891	0.533
FaceFour	0.909	0.898	0.864	0.864	0.909	0.784	0.886	0.852	0.818	0.943	0.807
FacesUCR	0.924	0.908	0.926	0.921	0.811	0.769	0.854	0.920	0.926	0.971	0.821
GunPoint	0.880	0.913	0.893	0.947	0.887	0.913	0.873	0.953	0.927	0.973	0.720
ItalyPowerDemand	0.948	0.955	0.935	0.955	0.948	0.955	0.948	0.948	0.932	0.944	0.881
Lightning7	0.589	0.712	0.616	0.740	0.548	0.575	0.521	0.753	0.658	0.753	0.548
MedicalImages	0.699	0.747	0.682	0.676	0.653	0.684	0.628	0.711	0.696	0.741	0.507
MiddlePhalanxOutlineAgeGroup	0.539	0.519	0.519	0.481	0.519	0.519	0.545	0.519	0.494	0.494	0.422
MoteStrain	0.903	0.866	0.858	0.872	0.859	0.879	0.908	0.797	0.881	0.855	0.550
Plane	1.000	1.000	1.000	1.000	1.000	0.962	1.000	1.000	1.000	1.000	0.990
ProximalPhalanxOutlineAgeGroup	0.810	0.785	0.815	0.795	0.800	0.785	0.805	0.805	0.727	0.790	0.678
SonyAIBORobotSurface1	0.750	0.696	0.815	0.689	0.775	0.696	0.734	0.681	0.785	0.730	0.704
SonyAIBORobotSurface2	0.843	0.859	0.864	0.823	0.877	0.859	0.846	0.853	0.885	0.871	0.703
ToeSegmentation1	0.816	0.750	0.851	0.833	0.662	0.680	0.811	0.820	0.789	0.816	0.632
ToeSegmentation2	0.900	0.908	0.915	0.923	0.823	0.808	0.938	0.785	0.892	0.754	0.815
Wine	0.611	0.611	0.611	0.611	0.574	0.611	0.593	0.574	0.648	0.593	0.574
Average	0.813	0.802	0.809	0.807	0.788	0.767	0.802	0.794	0.804	0.798	0.687

**Table 4:** The accuracies of 5 shapelet-based models and 3 DTW variants on 20 datasets.

Dataset	LSCR_DTW_	SDT	STC4.5	SSC4.5	FS	WDTW	CID	DD_DTW_
ArrowHead	0.749	0.754	0.686	0.686	0.594	0.817	0.829	0.789
Beef	0.733	0.533	0.500	0.600	0.567	0.700	0.633	0.667
BeetleFly	0.800	0.750	0.600	0.700	0.700	0.700	0.750	0.650
CBF	0.991	0.939	0.881	0.979	0.940	0.997	0.999	0.997
ECGFiveDays	0.863	0.994	0.958	0.990	0.998	0.796	0.782	0.769
FaceFour	0.909	0.795	0.750	0.750	0.909	0.875	0.875	0.830
FacesUCR	0.924	0.645	0.662	0.631	0.706	0.920	0.895	0.904
GunPoint	0.880	0.947	0.893	0.913	0.947	0.980	0.927	0.980
ItalyPowerDemand	0.948	0.958	0.954	0.942	0.917	0.950	0.956	0.950
Lightning7	0.589	0.507	0.699	0.575	0.644	0.767	0.699	0.671
MedicalImages	0.699	0.516	0.487	0.638	0.624	0.737	0.742	0.737
MiddlePhalanxOutlineAgeGroup	0.539	0.474	0.448	0.825	0.545	0.519	0.513	0.539
MoteStrain	0.903	0.823	0.760	0.818	0.777	0.859	0.796	0.833
Plane	1.000	0.933	0.905	0.838	1.000	1.000	1.000	1.000
ProximalPhalanxOutlineAgeGroup	0.810	0.781	0.771	0.844	0.780	0.805	0.790	0.800
SonyAIBORobotSurface1	0.750	0.735	0.877	0.849	0.686	0.737	0.815	0.742
SonyAIBORobotSurface2	0.843	0.659	0.757	0.795	0.790	0.831	0.877	0.892
ToeSegmentation1	0.816	0.895	0.899	0.882	0.956	0.794	0.737	0.807
ToeSegmentation2	0.900	0.869	0.869	0.800	0.692	0.892	0.877	0.746
Wine	0.611	0.796	0.759	0.778	0.759	0.574	0.611	0.574
Average	0.813	0.765	0.756	0.792	0.777	0.813	0.805	0.794

**Table 5:** The classification accuracies of 10 deep learning models on 20 datasets.

Dataset	LSTM-FCN	MLP	FCN	ResNet	Encoder	MCNN	t-LeNet	MCDCNN	Time-CNN	TWIESN
ArrowHead	0.909	0.778	0.843	0.845	0.804	0.339	0.303	0.685	0.723	0.659
Beef	0.900	0.720	0.697	0.753	0.643	0.200	0.200	0.563	0.763	0.537
BeetleFly	0.950	0.870	0.860	0.850	0.745	0.500	0.500	0.580	0.890	0.730
CBF	0.998	0.872	0.994	0.995	0.947	0.332	0.332	0.820	0.957	0.890
ECGFiveDays	0.992	0.970	0.987	0.975	0.982	0.499	0.497	0.762	0.882	0.698
FaceFour	0.943	0.840	0.928	0.955	0.815	0.268	0.295	0.712	0.906	0.855
FacesUCR	0.929	0.833	0.946	0.955	0.874	0.153	0.143	0.756	0.869	0.644
GunPoint	1.000	0.927	1.000	0.991	0.936	0.513	0.493	0.867	0.932	0.961
ItalyPowerDemand	0.963	0.954	0.961	0.963	0.965	0.500	0.499	0.955	0.955	0.880
Lightning7	0.836	0.630	0.827	0.845	0.625	0.310	0.260	0.534	0.651	0.664
MedicalImages	0.801	0.721	0.779	0.770	0.734	0.514	0.514	0.640	0.676	0.649
MiddlePhalanxOutlineAgeGroup	0.813	0.531	0.553	0.569	0.579	0.188	0.571	0.585	0.566	0.581
MoteStrain	0.939	0.858	0.937	0.928	0.840	0.508	0.539	0.765	0.882	0.785
Plane	1.000	0.978	1.000	1.000	0.976	0.130	0.134	0.965	0.965	1.000
ProximalPhalanxOutlineAgeGroup	0.893	0.856	0.831	0.853	0.844	0.488	0.488	0.838	0.828	0.844
SonyAIBORobotSurface1	0.982	0.672	0.960	0.958	0.743	0.443	0.429	0.653	0.687	0.638
SonyAIBORobotSurface2	0.978	0.834	0.979	0.978	0.839	0.594	0.617	0.774	0.841	0.697
ToeSegmentation1	0.983	0.583	0.961	0.963	0.659	0.505	0.526	0.490	0.595	0.865
ToeSegmentation2	0.931	0.745	0.880	0.906	0.795	0.632	0.815	0.443	0.738	0.842
Wine	0.870	0.565	0.587	0.744	0.500	0.500	0.500	0.500	0.517	0.759
Average	0.930	0.787	0.876	0.890	0.792	0.406	0.433	0.694	0.791	0.759

## Data Availability

The time series data used to support the findings of this study have been deposited in the UEA and UCR Time Series Classification Repository (http://www.timeseriesclassification.com).
